# BNT162b2 vaccine induced variant-specific immunity, safety and risk of Omicron breakthrough infection in children aged 5 to 11 years: a cohort study

**DOI:** 10.1038/s41598-023-44565-x

**Published:** 2023-10-13

**Authors:** Chee Fu Yung, Nina Le Bert, Kai Qian Kam, Seyed Ehsan Saffari, Chee Wah Tan, Yun Yan Mah, Jinyan Zhang, Aileen Ying-Yan Yeoh, Feng Zhu, Smrithi Hariharaputran, Chia Yin Chong, Antonio Bertoletti, Linfa Wang

**Affiliations:** 1 Infectious Disease Service, Department of Paediatrics, KK Women’s and Children’s Hospital, 100 Bukit Timah Road, Singapore, 229899 Singapore; 2 Duke-National University of Singapore Medical School, Singapore, Singapore; 3 Lee Kong Chian School of Medicine, Nanyang Technological University, Singapore, Singapore; 4 Programme in Emerging Infectious Diseases, Duke-NUS Medical School, Singapore, Singapore; 5 Yong Loo Lin School of Medicine, National University of Singapore, Singapore, Singapore; 6 Centre for Quantitative Medicine, Duke-NUS Medical School, National University of Singapore, Singapore, Singapore

**Keywords:** Diseases, Infectious diseases, Vaccines

## Abstract

There is little information on BNT162b2 vaccine-induced variant-specific immunogenicity, safety data and dynamics of breakthrough infections in pediatric populations. We addressed these questions using a prospective two dose BNT162b2 (10 mcg) vaccination cohort study of healthy children 5–11 years in Singapore. Follow up included blood samples at scheduled visits, daily vaccination symptom diary and confirmation of SARS-CoV-2 infection. Surrogate virus neutralization test (sVNT) and spike-specific T cell responses against SARS-CoV-2 variants were performed. The mean age of 127 participants was 8.27 years (SD 1.95) and 51.2% were males. The median sVNT level against original variant after 1 dose and 2 dose vaccination was 61.4% and 95.1% respectively (p < 0.0001). Neutralizing antibodies against the Omicron variant was the lowest, median 22.4% (IQR 16.5–30.8). However, T cell IFN-γ cytokine response against Omicron variant was high and remained so about 4 months after vaccination. Fever rate increased significantly from 4% (dose 1) to 11.5% (dose 2). The risk of Omicron breakthrough infection decreased by 7.8% for every 1% increase in sVNT inhibition level measured after dose 2 vaccination. BNT162b2 vaccines were safe, induced good T cell responses but poor neutralizing antibodies against Omicron in children. Low neutralizing antibody levels post-vaccination was predictive of subsequent breakthrough infection.

## Introduction

Coronavirus disease 2019 (COVID-19) is usually mild in children compared to adults and the elderly^1,2^. However, some children can develop severe disease requiring hospitalisation, oxygen supplementation and intensive care support^3^. Children with COVID-19 are also at risk of multisystem inflammatory syndrome in children (MIS-C) which can occur as a delayed response involving broad autoantibody production after recovery from the initial SARS-CoV-2 infection^4^.

Vaccinations and additional boosters have been proven to be effective against symptomatic COVID-19 and severe disease during the initial phase of the pandemic before the emergence of the Omicron variant^5^. In a phase 3 clinical trial, children aged 5 to 11 years who received 2 doses of 10 µg BNT162b2 vaccine (Pfizer/BioNTech) had similar antibody responses as adolescents aged 16 to 25 years who received 30 mcg^6^. Vaccine effectiveness was found to be 90.7% and subsequently, two doses of 10 mcg BNT162b2 given 21 days apart were licensed by the FDA for use in children 5 to 11 years. However, real-world effectiveness data of 2 or 3 doses of BNT162b2 against symptomatic SARS-CoV-2 infection have ranged from about 25% to 52% with the emergence of Omicron variants such as BA.1, BA.2, BA.4, BA.5 and XBB^7,8^. To date, immunological markers or correlates of protection which can reliably predict vaccine effectiveness against SARS-CoV-2 infection remain elusive.

In this study, we report SARS-CoV-2 variant-specific neutralizing antibody and T cell responses as well as the safety profile of two dose 10 mcg BNT162b2 vaccine given 21 days apart in a multi-ethnic cohort of children aged 5 to 11 years old in Singapore. We also investigated the association between neutralizing antibody levels post-vaccination and risk of breakthrough Omicron variant infection.

## Results

A total of 150 healthy children were recruited into the study. For the immune response analysis, 23 were excluded due to serological evidence of previous COVID-19 infection, had confirmed SARS-CoV-2 infection before completion of vaccination or visit 3, or did not complete visit 4. The remaining 127 patients had a mean age of 8.27 years (SD: 1.95) and 51.2% (n = 65) were males (Table 1). Ethnic distribution was 74.0% Chinese, 7.1% Malay, 5.5% Indian and 13.4% others (Eurasians, Caucasians, etc.).

**Table 1:** Demographics of participants.

	Overall	Not infected	Infected	p value
	(N = 127)	(N = 43)	(N = 84)	
Age on 1st dose				0.608
Mean (SD)	8.27 (1.95)	8.40 (2.04)	8.20 (1.91)	
Median [min, max]	8.00 [5.00, 11.0]	9.00 [5.00, 11.0]	8.00 [5.00, 11.0]	
Gender				0.915
Female	62 (48.8%)	22 (51.2%)	40 (47.6%)	
Male	65 (51.2%)	21 (48.8%)	44 (52.4%)	
Race				0.993
Chinese	94 (74.0%)	34 (79.1%)	60 (71.4%)	
Indian	7 (5.5%)	2 (4.7%)	5 (6.0%)	
Malay	9 (7.1%)	2 (4.7%)	7 (8.3%)	
Others	17 (13.4%)	5 (11.6%)	12 (14.3%)	

In the cohort (n = 127), 66.1% of the children reported confirmed SARS-CoV-2 infection by ART or PCR after completion of 2-dose vaccination during the follow-up period. There were no significant differences in the age, gender and ethnic distributions between the infected and uninfected participants. All the children infected with COVID-19 had mild disease and did not require hospital admission.

### Neutralizing antibody response (sVNT) in children with two dose BNT162b2 vaccine

**Figure 1:**
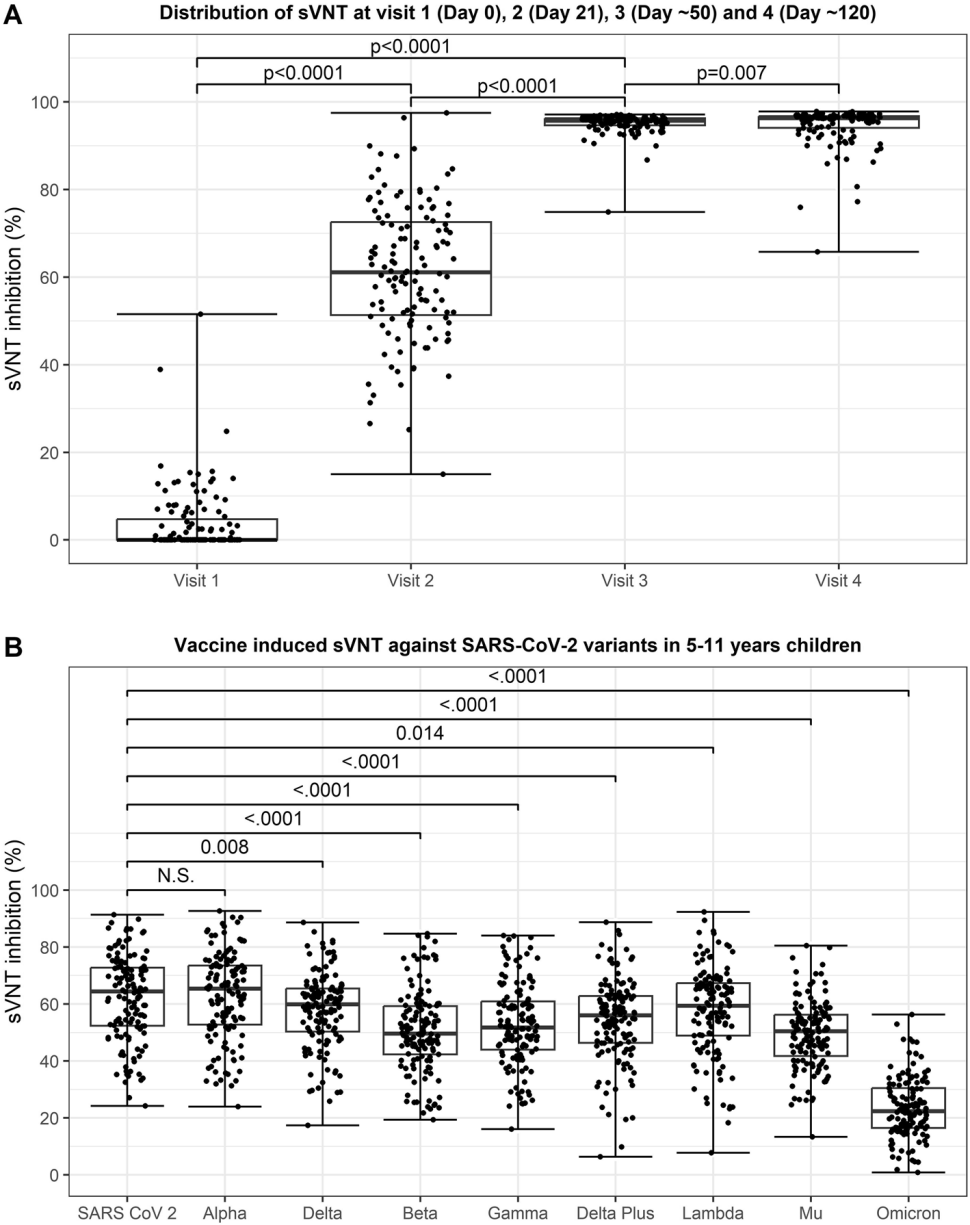
Surrogate virus neutralization test (sVNT) after vaccination in children 5–11 years. (**A**) Distribution of sVNT at visit 1 (Day 0), 2 (Day 21), 3 (Day ~50) and 4 (Day ~120). (**B**) Vaccine induced sVNT against SARS-CoV-2 variants after the second dose at Visit 3 (Day ~50) in 5–11 years children.

Figure 1 shows the distribution of sVNT (%) over time from vaccination dose 1 to about 4 months. There was a statistically significant increase in median inhibition from dose 1 to dose 2 (p < 0.0001). The mean and median inhibition levels after 2 dose vaccination (Visit 3) was 95.1% and 95.7% respectively (Table S1). Subsequently at about 4 months after start of vaccination, the mean and median inhibition levels remained high at 94.7% and 96.3% respectively.

Using a multiplex surrogate virus neutralization assay, we found that vaccination elicited neutralizing antibodies against ancestral SARS-CoV-2, Alpha, Delta, Beta, Gamma, Delta plus, Lambda, Mu and Omicron variants (Fig. 1B). The mean inhibition level against the Alpha variant was highest at 65.4% (IQR 52.7–73.5) followed closely by ancestral SARS-CoV-2 variant at 64.4% (IQR 52.3–72.7). However, the level of neutralizing antibody were lower for the Delta, Beta, Gamma, Delta plus, Lambda and Mu variants. Neutralization against the Omicron variant was the lowest with a median level of 22.4% (IQR:16.5 to 30.8).

### T cell responses in children with two-dose BNT162b2 vaccine

**Figure 2:**
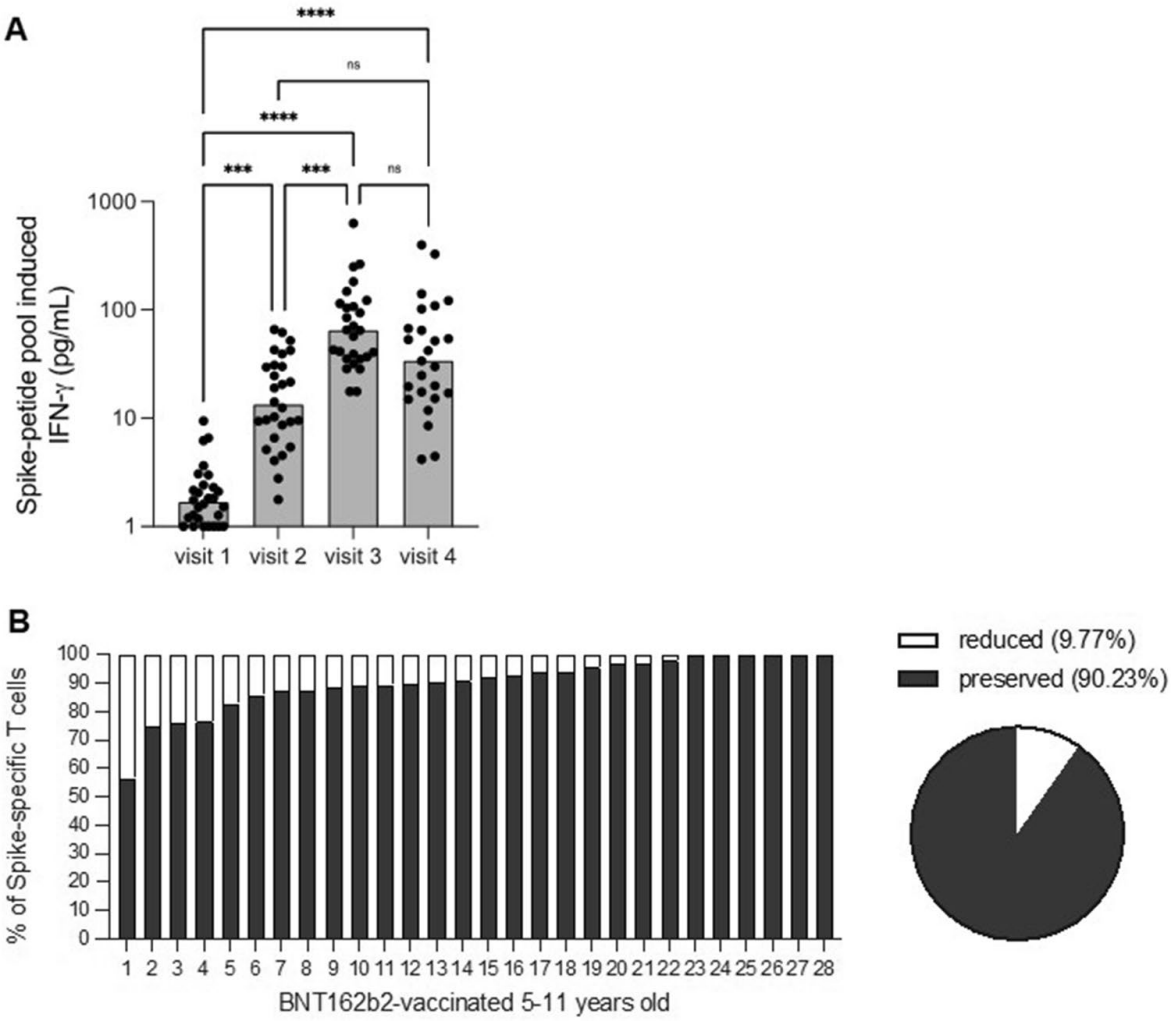
Spike-specific T cell responses in vaccinated children. (**A**) IFN-γ secretion profile of whole-blood cultures stimulated with Spike protein peptide pool compared at different time points of paired samples from vaccinated individuals (n = 28). The limit of detection for IFN‐γ = 1.7 pg/ml. Values below limit of detection levels were plotted as 1. The mean values of each group are represented by a red line and were at visit 1, 2, 3 and 4: 1.7 pg/ml, 13.3 pg/ml, 64.8 pg/ml and 33.8 pg/ml of IFN‐γ, respectively. (**B**) PBMC of 5–11 years old children (n = 28) were tested on day 49 for their ability to respond to the Omicron variant of SARS-CoV-2 using IFN-γ ELISpot assays. Bars represent each individual; pie chart shows the mean reduction in the T cell response to Omicron.

Prior to vaccination, whole blood cultures from some children in the cohort were found to be positive for SARS-CoV-2 Spike-peptide mediated IFN-γ cytokine response (Fig. 2A). Following receipt of the first and second dose vaccination, IFN-γ levels were significantly boosted. No significant drop in IFN-γ cytokine response was detected at about 4 months after initiation of vaccination. Vaccine-induced T cell immunity was largely preserved against Omicron in all tested children (mean = 9.77% inhibition; Fig. 2). Only 4 of the 28 children had reduced T cell responses by more than 20%.

### Neutralizing antibody levels post-vaccination predicts risk of Omicron variant infection

**Figure 3:**
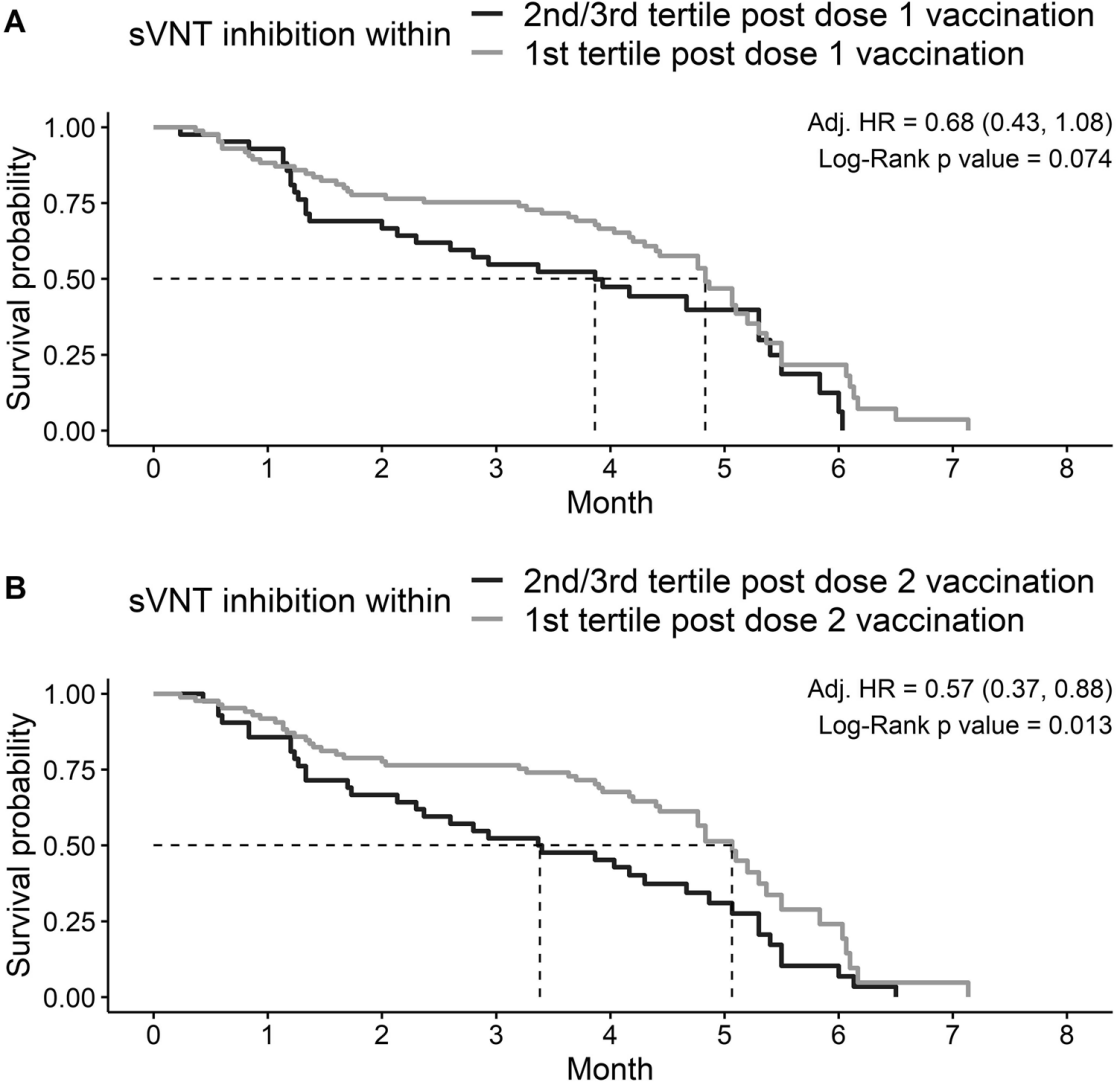
Kaplan–Meier plot of neutralizing antibodies (sVNT) post dose 1 (**A**) and dose 2 (**B**) versus time to SARS-CoV-2 infection.

After one dose at visit 2, the sVNT inhibition level of 54% was the cut-off between the top tertiles (1st tertile) and bottom two tertile (2nd and 3rd tertiles). After dose 2 at visit 3, the cut-off sVNT inhibition level was 95% for the same groupings. As shown in the Kaplan Meier plots (Fig. 3), the median time to Omicron infection in participants with sVNT inhibition level > 95% and < 95% after 2 doses of BNT162b2 were 5.1 months (95% CI 4.8 to 5.5 months) and 3.4 months (95% CI 2.3 to 4.9 months), respectively.

Cox regression model adjusted for age and gender found that individuals with sVNT inhibition levels > 54% after one dose of BNT162b2 vaccine had a lower risk of Omicron infection compared to those with inhibition levels < 54% but the difference was not statistically significant (HR 0.68, 95% CI 0.43–1.08). However, adjusted cox regression analysis of neutralizing antibody levels at visit 3 after the second dose was found to predict the risk of subsequent Omicron infection. Those with sVNT inhibition levels > 95% were significantly less likely to be infected during the Omicron wave compared to those with inhibition levels < 95%. (HR 0.57, 95% CI 0.37–0.88). Overall, after adjusting for age and gender, the risk of infection decreased by 1.2% and 7.8% for every 1% increase in sVNT inhibition level at Visit 2 (after dose 1) and Visit 3 (after dose 2), respectively.

### Adverse events following immunization

Vaccine diary data from all 150 children were included in the vaccine safety analysis. Approximately 70% of participants reported at least one adverse event within the 7 days post-vaccination (Table 2). The most common side effect reported was pain at the injection site (67.3% after 1st dose, 62.8% after 2nd dose). Fever was reported in 4% of participants after dose 1 and in 11.5% after dose 2. The higher rate of fever after dose 2 was statistically significant (p = 0.012). There was no obvious association between age and any adverse events following immunization rates (Supplementary Fig. S1). None of the reported adverse event following immunization required medical care or hospitalization. There were no reported severe adverse reactions e.g. myocarditis, anaphylaxis, etc. post-vaccination.

**Table 2:** Safety data of BNT162b2 vaccine in children 5 to 11 years.

Side effect	1st dose		2nd dose		p value
	Number	%	Number	%	
Fever	6	4	17	11.5	0.012
Rash, lip/eye/face swelling	3	2	5	3.4	NS
Pain at injection site	101	67.3	93	62.8	NS
Medical attention	0	0	0	0	NS
Redness/swelling at injection site	18	12	21	14.2	NS
Headache/body ache	29	19.3	22	14.9	NS
Other reactions	15	10	11	7.4	NS
Any side effect	110	73.3	100	67.6	NS

*NS* not statistically significant.

## Discussion

In our cohort of children 5 to 11 years old, two doses of 10 mcg BNT162b2 vaccine given 21 days apart generated robust neutralizing antibodies against ancestral SARS-CoV-2 which remained high up to about 4 months after initiation of vaccination. Although neutralizing antibodies against other VOCs were also generated, the level of neutralizing antibodies specifically against Omicron variant was very low. However, we confirmed that two-dose vaccination with BNT162b2 induced spike-specific T cell responses including against the Omicron variant in young children. A sVNT inhibition level < 95% after dose 2 was found to be significantly predictive of subsequent breakthrough infection (HR 0.57, 95% CI 0.37 to 0.88). All children in our cohort who had breakthrough SARS-CoV-2 infection had mild disease and none required hospitalizations. The vaccine had a good safety profile in our multi-ethnic pediatric cohort and there were no reported adverse events that required hospitalization.

Immune response data to complement vaccine effectiveness data are important to better understand the dynamics of protection in different age groups. Similar to adult studies, we showed that children 5 to 11 years old were able to generate reasonable levels of neutralizing antibodies as well as spike-specific T cells after a single dose of 10 mcg BNT162b2^9^. There was a significant boost to much higher levels of neutralizing antibody levels and T cell responses after receipt of the second dose 21 days later. We found very little waning of vaccine-induced neutralizing antibodies approximately 4 months after initiation of vaccination in our pediatric cohort (median sVNT at about 4 m = 96.3%). In contrast, Renia et al. reported median sVNT of 89.9% and 67.4% at 3 months and 6 months post-vaccination, respectively in a study of adults and elderly^10^. In terms of T cell memory response, we did not detect any significant decrease in IFN-γ cytokine levels at about 4 months post-vaccination, similar to findings in adult/elderly cohort by Renia et al. Both, ours and Renia et al. samples were analysed by the same laboratories using the same techniques and assays. These contrasting findings highlight differences in humoral responses between children and adult/elderly. Vaccination policies or recommendations in children may need to be assessed separately from the adult/elderly.

Our study confirms the generation of neutralizing antibodies against a number of VOCs in children 5 to 11 years old following two doses of 10 mcg BNT162b2 given 21 days apart. However, the level of neutralizing antibodies against Omicron variant compared to other VOCs was significantly lower. These findings echo similar adults based studies showing the high resistance and evasion properties of Omicron variant against vaccine-induced neutralizing antibodies^11^. A third dose was found to mitigate although it was unable to fully overcome this effect in adult studies, but similar immunogenicity data in children remain limited^10^. Importantly, we confirmed that vaccine-induced T cell response in children was able to able to respond to the highly mutated SARS-CoV-2 variant B.1.1.529 (Omicron). T cells are able to recognize all peptide epitopes of the SARS-CoV-2 Spike protein and hence are less susceptible to antibody escape mutations of the Omicron variant^12^. Our results concur with real-world vaccine effectiveness studies showing continued protection against severe COVID-19 in children despite reduced protection against SARS-CoV-2 infection^7^.

To date data on immune dynamics of protection against SARS-CoV-2 especially in children remain scarce. We found that the level of neutralizing antibodies measured at Visit 3 about 1 month after receipt of dose 2 could potentially be used to predict risk of subsequent breakthrough infection during the Omicron wave. As shown in the Kaplan–Meier plots (Fig. 3), there were clear differences in terms of duration of clinical protection in children above and below sVNT of 95%. We found that the median duration of protection in our pediatric cohort was 3.4 months for those with sVNT level < 95% versus 5.1 months for those with sVNT level > 95% measured about a month after dose 2. After adjustment for age and sex, cox regression analysis confirmed that children with sVNT level > 95% were 43% (HR 0.57, 95% CI 0.37–0.88) less likely to be infected compared to those with sVNT level < 95%. Every 1% increase in sVNT level post-dose 2 reduced the risk of breakthrough infection by 7.8% in our pediatric cohort. We observed a similar trend (HR 0.68, 95% CI 0.43–1.08) with a cut-off of sVNT level 54% for neutralizing antibodies measured after dose 1 but this was not statistically significant. Taken together, these findings concur with adult based studies which have shown a direct correlation between neutralizing antibody levels and risk of breakthrough infections^13^.

The safety profile of COVID-19 vaccines in children is crucially important in view of their lower risk of severe disease. The common adverse event reported in our cohort was pain at the injection site at a rate of 67.3% after 1st dose and 62.8% after 2nd dose. Fever was reported in about 4% of participants after dose 1 and this significantly increased to 11.5% after dose 2. Importantly, none of the adverse events reported required hospitalization. Overall, the risk of adverse events was lower than observed in adolescents and adults who received a higher antigen dose of BNT162b2 at 30 mcg^14^.

A limitation of our study was that we only measured neutralizing antibodies to the receptor binding domain of SARS-CoV-2. However, neutralizing antibodies have been shown to be an important component of the immune system that correlates with protection against SARS-CoV-2 infection^13^. We used an arbitrary tertile stratification of neutralizing antibody levels in the absence of known cut-off as the correlate of protection. Our data provided estimates of risk of infection over 6 months during the Omicron wave. Whether the findings are valid over a longer duration or against other VOCs requires further research. However, vaccine estimates of COVID-19 vaccines against Omicron subtypes have remained fairly similar at around 50% to date^8^. SARS-CoV-2 infection was based on both ART and/or PCR testing results reported by parents/guardian. During this period, Singapore had very strict public health contact tracing measures in place and ART test kits were widely available including free distribution to every household. Therefore, case misclassification was unlikely but we cannot exclude the possibility of missed fully asymptomatic infections with unknown contact to a case.

## Conclusions

BNT162b2 vaccines were immunogenic and safe in children 5 to 11 years old in our multi-ethnic cohort study. Two dose vaccination induced good levels of neutralizing antibodies against most VOCs except Omicron. Crucially, T cell immunity against Omicron variant was found to be high. Neutralizing antibodies and T cell memory remained elevated about 4 months after initiation of vaccination in children. We found that the risk of Omicron breakthrough infection decreased by 7.8% for every 1% increase in sVNT inhibition level measured after dose 2 vaccination.

## Methods

### Setting and participants

This is a prospective cohort study from December 2021 to July 2022 involving healthy children aged 5 to 11 years in Singapore. Patients with immunocompromised conditions (e.g. with oncological diagnosis, on immunosuppressive therapies) and previous known COVID-19 infection were excluded from this study. During the study period, Singapore experienced its first Omicron wave from January to April, 2022, driven by the Omicron (BA.1 and BA.2) variant^15^.

The study was approved by Singhealth Centralised Institutional Review Board (2021/2463). Written informed consent/assent was obtained from all participants and parents/guardians. All experiments were performed in accordance with the relevant guidelines and regulations.

### Vaccination, follow up and vaccine diary

Participants received two doses of 10 mcg of BNT162b2 vaccine given 21 days apart. Demographic data was collected from the participants. Blood samples were collected during the following visits: Visit 1: day of 1st dose of vaccine (Day 0) prior to vaccination; Visit 2: day of 2nd dose of vaccine (Day 21) prior to administration of vaccine; Visit 3: Day 49 (− 7/ + 30 days) post-dose 2; Visit 4: Day 120 (− 30/ + 30 days). The study participants were scheduled for follow-up for 2 years from recruitment but in this paper we will present the data up to visit 4. Participants had to fill up a daily symptom diary for 7 days post-vaccination to assess for vaccine safety. There was a monthly follow-up phone call made to participants to evaluate if the participants had COVID-19 infection diagnosed via self-administered antigen rapid test (ART) or healthcare performed polymerase chain reaction (PCR) test post vaccination. If a participant reported SARS-CoV-2 infection, the date of infection was based on the first recorded day of positive ART/PC. All COVID-19 infections were assumed to be Omicron variants which was the predominant circulating strain during the study period.

### Neutralization antibody levels

We used the surrogate virus neutralisation test (sVNT) for SARS-CoV-2 (cPass, Genscript) according to the manufacturer’s instructions. Briefly, 1:20 diluted serum samples were pre-incubated with equal volume of HRP-conjugated SARS-CoV-2 RBD for 30 min at 37 °C prior addition to the ACE2-coated ELISA plate for additional 15 min at 37 °C. After five washes, the signal was developed and the absorbance reading at 450 nm was acquired using Cytation-5 microplate reader. Multiplex sVNT was performed as described previously with some modifications^16^. Briefly, 1:50 diluted serum were pre-incubated with equal volume of SARS-CoV-2 and variants (Alpha, Delta, Beta, Gamma, Delta plus, Lambda, Mu and Omicron BA.1) RBD-coated microsphere (600 microspheres/antigen/reaction) for 15 min at 37 °C, followed by addition of PE-conjugated ACE2 (2000 ng/ml) for 15 min at 37 °C. The mean fluorescence intensity (MFI) was acquired using MagPix platform (Luminex). The percentage inhibition was calculated as follows:

$$ \% {\text{ Inhibition }} = \, \left( {{1} - \, \left( {{\text{MFI}}\;{\text{ sample}}/{\text{MFI}}\;{\text{ negative }}\;{\text{control}}} \right)} \right) \, \times { 1}00\% $$

### T cell immune response

We evaluated longitudinal spike-specific T cell responses in a sub-cohort of 28 vaccinated children. To measure spike-peptide induced cytokines, 320 µl of fresh whole blood was mixed with 80µl RPMI and stimulated with SARS-CoV-2 peptides (2 µg/ml; Hyris Ltd. Spike-A) or DMSO. After 15 h, the culture supernatant was quantified for IFN-γ using an ELLA machine (ProteinSimple). Cytokine level present in DMSO controls was subtracted from the corresponding peptide pool-stimulated samples^17^.

To investigate the impact of Omicron variant on Spike-specific T cells, PBMCs were stimulated with 3 peptides pools (Hyris): (1) “SP-MP” pool covering the entire ancestral Spike protein; (2) “Spike Hotspot-Ancestral” pool covering the variable regions in Omicron; (3) “Spike Hotspot-Omicron” pool consisting of the Omicron-derived Spike peptides covering the same region. Total spot forming units (SFU) formed against the entire Omicron variant Spike was derived using the equation below:

$$ {\text{SFU}}_{{{\text{Total}}\;{\text{Omicron}}\;{\text{Spike}}}} = {\text{ SFU}}_{{\text{SP-MP}}} {-}{\text{ SFU}}_{{{\text{Spike }}\;{\text{Hotspot-Ancestral}}}} + {\text{ SFU}}_{{{\text{Spike }}\;{\text{Hotspot-Omicron}}}} $$

From this, % inhibition due to variation in Omicron variant Spike sequences was quantified using the equation below:

$$\mathrm{Inhibition}=\frac{\mathrm{SFUSP}-\mathrm{MP}-\mathrm{ SFUTotal Omicron}-\mathrm{Spike}}{\mathrm{SFUSP}-\mathrm{MP}}$$

### Statistical analysis

Statistical analysis was performed using SAS software version 9.4 for Windows (Cary, NC: SAS Institute Inc.). Statistical significance was set at p < 0.05. Distribution of demographic variables were presented as mean, median, standard deviation, minimum and maximum for age at 1st dose and frequency and percent for sex and race variables for the entire cohort as well as by the infection status. sVNT% neutralizing antibody level results were presented as box-plot for each time point, and pairwise comparisons between the time points were performed using Mann–Whitney *U* test. For T cell analysis, the IFN-γ levels were compared using one-way ANOVA followed by Tukey’s multiple comparison tests. Safety data was presented as frequency and percent and were compared between 1st and 2nd dose using McNemar test.

To investigate the association between neutralizing antibody levels which is represented by sVNT and breakthrough infection risk, sVNT levels (%) at visit 2 (post-dose 1) and visit 3 (post-dose 2) were stratified into tertiles (top tertile = 1st tertile, middle tertile = 2nd tertile and bottom tertile = 3rd tertile). Kaplan–Meier plots were generated for the time to SARS-CoV-2 infection between sVNT levels of 1st tertile vs 2nd/3rd tertile at visit 2 and visit 3. Differences between 1st tertile vs 2nd/3rd tertile were assessed via log-rank test. Finally, Cox regression analysis was performed and the results were presented as adjusted hazard ratio (HR) and 95% confidence interval (CI) controlling for age and sex. The adequacy of the Cox regression model was assessed via checking the proportional hazards assumption by cumulative sums of martingale residuals over follow-up times or covariate values. Firth’s penalised likelihood approach was applied to reduce bias in parameter estimates.

### Supplementary Information

Supplementary Information.

## Data Availability

All requests for data will be reviewed by the corresponding author to determine confidentiality obligations. Enquiries regarding data availability should be directed to the corresponding author.
