# Supplementary material for: BNT162b2 vaccine induced variant-specific immunity, safety and risk of Omicron breakthrough infection in children aged 5 to 11 years: a cohort study
Source: Sci Rep. 2023 Oct 13;13:17337. doi: 10.1038/s41598-023-44565-x (PMC10575958; doi:10.1038/s41598-023-44565-x)
Supplement: Supplementary file 1 — Supplementary Information. [file 41598_2023_44565_MOESM1_ESM.docx]

**Appendix/Supplementary**

**Supplementary Table S1: sVNT % level (mean, median, IQR and range) at each study visit.**

|  | **Day 0**  **(Visit 1)** | **Day 21**  **(Visit 2)** | **Day 49**  **(Visit 3)** | **Day 120 (visit 4)** |
| --- | --- | --- | --- | --- |
|  |  |  |  |  |
| Mean (SD) | 3.57 (7.29) | 61.4 (15.4) | 95.1 (2.40) | 94.7 (4.57) |
| Median [IQR] | 0 [4.74] | 61.1 [21.3] | 95.7 [1.60] | 96.3 [2.66] |
| Q1-Q3 | 0-4.74 | 51.3-72.6 | 94.7-96.3 | 94.1-96.7 |
| Min, Max | 0, 51.6 | 15.0, 97.5 | 74.9, 97.1 | 65.7, 97.8 |

**Supplementary Figure S1: Any adverse event following immunisation rates by yearly age groups and vaccine dose.**

**
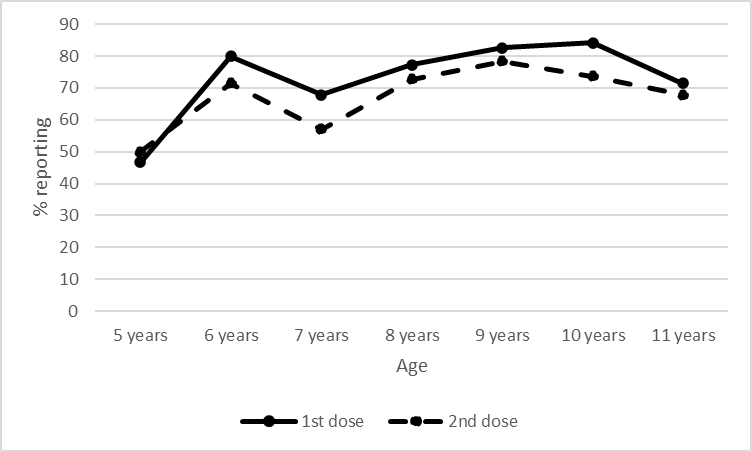
**
